# Sodium acetate and sodium butyrate attenuate diarrhea in yak calves by regulating gut microbiota and metabolites

**DOI:** 10.1016/j.heliyon.2024.e26564

**Published:** 2024-02-24

**Authors:** Qinghui Kong, Xiushuang Chen, Yang Liu, Farah Ali, Asif Idrees, Farid Shokry Ataya, Zhenda Shang, Kun Li

**Affiliations:** a Key Laboratory of Clinical Veterinary Medicine in Tibet, Tibet Agriculture and Animal Husbandry College, Linzhi, 860000, Tibet, China; b Institute of Traditional Chinese Veterinary Medicine, College of Veterinary Medicine, Nanjing Agricultural University, Nanjing, 210095, China; c MOE Joint International Research Laboratory of Animal Health and Food Safety, College of Veterinary Medicine, Nanjing Agricultural University, Nanjing, 210095, China; d Institute of Animal Husbandry and Veterinary Medicine, Tibet Academy of Agricultural and Animal Husbandry Sciences, Lhasa, 850000, China; e Faculty of Veterinary and Animal Sciences, The Islamia University of Bahawalpur, 63100, Pakistan; f KBCMA, College of Veterinary and Animal Sciences, Narowal, Pakistan; g Department of Biochemistry, College of Science, King Saud University, PO Box 2455, Riyadh, 11451, Saudi Arabia

**Keywords:** Diarrhea, Yak, Sodium acetate, Sodium butyrate, Microbiota, SCFAs

## Abstract

Diarrhea is a severe issue in calves that causes fertility problems and economic issues worldwide. Sodium acetate/sodium butyrate (SA/SB) alleviates diarrhea in mice; however, little information is available about the preventive effect of SA/SB on diarrheic yak calves living on the Tibet plateau. Yak calves (n = 19) of age ≥4 months and weight 37 ± 2 Kg were randomly divided into control (C, n = 10) and supplement groups (S, n = 9). Yaks belonging to the supplement group were given sodium butyrate (10 g/kg) and sodium acetate (5 g/kg) for 28 days, along with normal feed, seasonal grasses, pasture, and water. The blood and fecal samples from yak calves were collected for assessment of antioxidant capacity, inflammatory cytokines, microbiome, and short-chain fatty acids (SCFAs) concentration analysis. Results of this study revealed that a lower diarrhea rate, higher weight, and net weight gain were recorded in yaks belonging to group S supplemented with SA/SB. Similarly, increased antioxidant capacity with higher levels of T-AOC, SOD, and GSH-px and decreased inflammatory reactions by decreasing both TNF-α and IL-1β concentrations were recorded in yaks of group S. The concentration of SCFAs was significantly higher (*p* < 0.05) in yaks from group S than group C. Microbiome analysis revealed that 8 phyla and 54 genera were significantly different (*p* < 0.05) in both yak groups, with increased probiotics (Akkermansia, Oscillospira), SCFAs producing genera (Oscillospira, ASF356, Anaerosporobacter and Phascolarctobacterium), and decreased inflammatory related genus (Flavonifractor, Fournierella) and harmful bacteria (Oscillibacter, Achromobacter) in group S. In conclusion, the results demonstrated that SA and SB could decrease diarrhea rates in yak calves on the plateau via increasing antioxidant ability and SCFAs, while decreasing inflammatory responses in yaks by moderating gut microbiota. The current results provide new insights for the prevention and treatment of diarrhea in yaks.

## 1 Introduction

*Bos grunniens* (Yak) is an important animal on high plateaus in countries around the Qinghai-Tibet Plateau including China, Mongolia, and adjoining areas of Pakistan [1]. It is an indigenous bovine species providing meat, milk, fuel, fur, and means of transportation for the local herdsmen [2,3]. There are approximately 14 million yaks in China, which accounts for 90% of the world total yak population [4]. In recent years, diarrhea was reported to occur frequently in yaks with high mortality and weight losses, which seriously hindered yak breeding in the plateau regions [[4], [5], [6], [7]].

Diarrhea is a general pathological condition in calves, prompting economic constraints throughout the world [1,8]. It is reported that recovered animals from diarrhea usually exhibit subsequent growth impairment, which afterward reduces breeding and causes loss to the livestock industry [1,9]. In the United States, calf deaths due to diarrhea account for 56.4% of pre-weaned dairy deaths [10]. Diarrhea is associated with various kinds of infectious and non-infectious etiological factors [11]. Various practices, including herd management, better nursing, feeding, nutrition, and utilization of biopharmaceuticals are implemented, but it is still an immense challenge to figure out diarrhea in calves [12]. Previous studies showed that diarrhea was mainly linked to intestinal dysbacteriosis [6,13]. It was also revealed in previous studies that gut microbiota imbalances occur in diarrheic yaks on the plateau [[14], [15], [16]].

The intestinal microbiota of animals is comprised of trillions of microbes, including archaea, viruses, bacteria, and eukaryotes [17], which show significant improvements to animal's health like improved digestion and absorption, increased vitamin synthesis, limited pathogen harboring, and modulation of immune functions [18,19]. Short-chain fatty acids (SCFAs) are produced by the host microbiota through anaerobic fermentation of dietary fibers [20], which can alter host metabolism, intestinal function, intestine homeostasis, and immunity [18]. SCFAs are composed of several acids including acetate, propionate, butyrate, etc., while acids with C2–C4 are the primary acids in the gut microbiome [21]. In a previous study, we discovered a lower abundance of SCFAs in diarrheic yaks [22,23], while supplemented SA/SB attenuated mice diarrhea induced by lipopolysaccharide [24]. However, limited information is known about the beneficial effect of SA/SB against diarrhea in yaks on the plateau. Therefore, the current study was conducted to explore the mitigation effect of sodium acetate and sodium butyrate supplementation on naturally occurring diarrhea in yaks.

## 2 Materials and methods

### 2.1 Experiment design

**Fig. 1:**
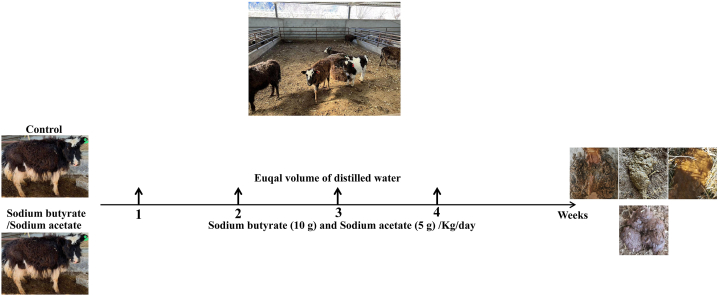
Experimental design through flow diagram of current study.

Yak calves (n = 19) of age ≥4 months and weight 37 ± 2 Kg were reared near a slaughter house in Nyingchi (with an average altitude of 3100 m), China for four weeks (28 days) during December 2022 to January 2023. The yaks were randomly divided into control (C, n = 10) and supplement groups (S, n = 9). The control group was provided normal feed, seasonal grasses, pasture, and water ad libitum. Besides providing normal feed, seasonal grasses, pasture, and water, the supplement group was given sodium butyrate (10 g/kg/day) and sodium acetate (5 g/kg/day) for 28 days, and the control group was given an equal volume of water. The SA and SB were supplemented orally with the normal feed. The body weights and diarrhea of calves were recorded on a weekly basis. Fresh samples of feces and blood from all yaks were collected at the end of the current experiment (Fig. 1).

### 2.2 Antioxidant capacity, inflammatory cytokines and NOS in serum of yak calves

The serums were separated from the collected blood samples of animals by centrifuging at 3500 g for 20 min, then kept at −20 °C for further analysis. Four inflammatory cytokines (IL-1β, IL-6, IL-10, and TNF-α) in yak calves were analyzed by using ELISA kits (Solarbio Life Science, China) according to manufacturer instructions and procedures. The serum levels of oxidation resistance indicators, including total antioxidant capacity (T-AOC), superoxide dismutase (SOD), glutathione peroxidase (GSH-px), and malondialdehyde (MDA), along with nitric oxide (NO) in calves were analyzed using commercial assay kits (Jiancheng Bioengineering Institute, China).

### 2.3 Yak gut microbiome analysis

Initially, six fecal samples of yaks from group C and group S, were utilized for total genomic DNA extraction following the instructions of GenElute™ fecal DNA separation kit (Sigma-Aldrich, Germany). Quantity and quality inspection of DNA products from yak calves were performed, and the 16S rRNA gene was amplified according to previously studies [23,24]. Later on, PCR products were purified and quantified for sequencing via the Illumina platform with MiSeq Reagent Kit v3 (Illumina, United States) as per the previous study [24].

Then raw sequence data from yak calves was processed through the QIIME2 system [25], then the obtained data was analyzed to produce an amplicon sequence variant (ASV) feature table for generating the taxonomy table by using the Greengenes database through the QIIME2 dada2 [26]. Diversities of alpha and beta were calculated by the core-diversity plugin in QIIME2 such as observed operational taxonomic units (OTUs), Chao1, Shannon, and Faith's to evaluate samples inside microbial diversity. Then, it was estimated through principal coordinate analysis [27], nonmetric multidimensional scaling [28], Qiime2 β analysis [29] and principal component analysis [30] to explore microbiota variation between two yak groups as per the previous study [31]. To reveal the distinguished different microbiota between group C and group S, the methods of LEfSe and DEseq2 were piloted [32,33].

Ultimately, microbiota function (KEGG ortholog, KEGG, metacyc pathway abundance, and enzyme abundance) and differences between the two yak groups were analyzed using PICRUSt [34].

### 2.4 Yak fecal SCFAs analysis

The concentration of SCFAs in fecal samples of ruminant calves was measured using gas chromatography (Agilent HP 6890 series, USA) according to the protocol adopted in the previous study [35].

### 2.5 Statistical analysis

The difference between the experimental groups were examined using ANOVA and Dunn's test through IBM SPSS (19.0) software. The data were presented as means ± SD, and statistical significance was noticed when *p* < 0.05.

## 3 Results

### 3.1 SA/SB increased body weights and decreased diarrhea score in yak calves

**Fig. 2:**
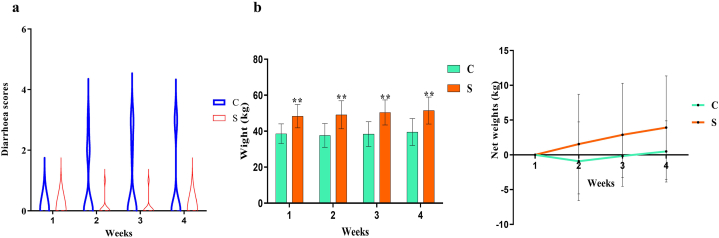
SA/SB supplementation increased in body weight and decreased diarrhea score in yak calves (a) diarrhea score, (b) body weight. **p < 0.01; data are presented as the mean ± SEM (n = 9).

In the present study, diarrhea in yaks was recorded for 4 weeks, and it was observed that an increased diarrheic trend was found in the control group compared to the supplement group (Table 1). Whereas, diarrhea score analysis showed that from the second to fourth weeks, the diarrhea score in control animals was the highest (Fig. 2a). The weight of yak calves in the S group was remarkably higher than that of control animals (*p* < 0.01), and the net weight was also higher in yaks in group S (Fig. 2b).

**Table 1:** Diarrhea rate in yak's calves at different weeks.

	Time Interval (weeks)			
Groups	1	2	3	4^a^
C (n = 10)	0 (0)	30% (3)	30% (3)	22.2 % (2)
S (n = 9)	0 (0)	11.1% (1)	11.1% (1)	0 (0)

a Died (n = 1).

### 3.2 Sodium acetate/sodium butyrate increased antioxidant ability and decreased inflammatory response in yak calves

**Fig. 3:**
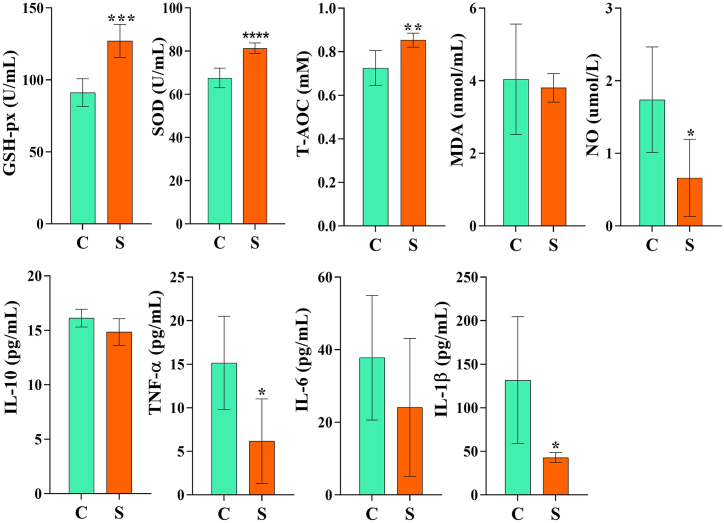
The effect of SA/SB on antioxidant capacity, inflammatory cytokines and NO. *p < 0.05, **p < 0.01, and ****p < 0.0001; data are presented as the mean ± SEM (n = 6).

The concentrations of GSH-px (*p* < 0.001), SOD (*p* < 0.0001), and T-AOC (*p* < 0.01) were obviously higher in yaks of group S compared to group C, while NO, TNF-α, and IL-1β were significantly (*p* < 0.05) lowered in group S compared to group C. Whereas, there was no significant difference (*p* > 0.05) for MDA, IL-10, and IL-6 between the groups (Fig. 3).

### 3.3 Effects of SA/SB feeding on microbiota structure and diversity of yak calves

**Fig. 4:**
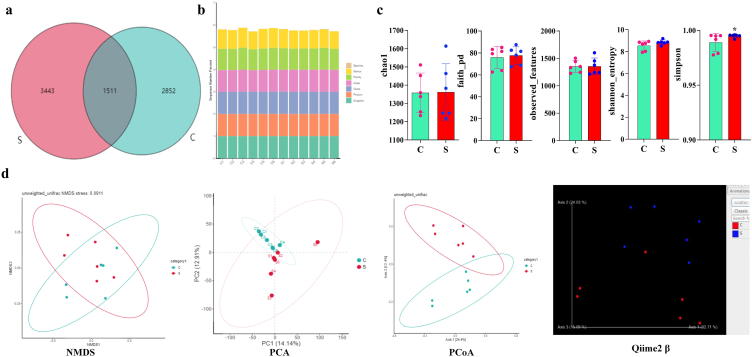
Effects of SA/SB feeding on microbiota structure and diversity of yak calves. (a) Venn map, (b) Annotation statistics diagram, (c) Alpha diversity index analysis, (d) Beta diversity analysis. *p < 0.05; data are presented as the mean ± SEM (n = 6).

A total of 778688 and 790068 inputs and 713878 and 725532 filtered sequences were generated in calves from groups C and S, respectively, in the current study (Table 2). These sequences were aligned to 4363 and 4954 ASVs in yaks in both groups, with 1511 shared ASVs (Fig. 4a). The total of 7806 ASVs were annotated to different taxa (Fig. 4b). Alpha diversity analysis found that there was no significant difference (*p* > 0.05) of chao1, faith_PD, observed_features, and Shannon_entropy between yak calves in groups C and S; however, Simpson was significantly (*p* < 0.05) higher in SA/SB supplemented yak's calves than the control group (Fig. 4c). Beta diversity analysis indicated that feeding sodium acetate and sodium butyrate extended the distance between yak calves, as shown by PCoA and Qiime2 β (Fig. 4d) (see Table 3).

**Table 2:** Yak fecal sequencing data analysis.

Sample ID	Input	Filtered	Denoised	Merged	Non-chimeric
C1	126874	114268	109879	83260	66978
C2	127033	114788	110734	83050	70271
C3	134246	123899	121447	104605	90228
C4	127110	117765	114644	100036	85766
C5	132600	123251	120834	101830	89076
C6	130825	119907	117553	103192	88383
S1	134223	124964	122443	78027	70368
S2	134538	125083	123281	112458	98580
S3	126853	113596	109134	80793	68871
S4	133222	119915	115603	73014	65003
S5	132072	122365	119682	76385	65353
S6	129160	119609	116034	86529	76457

**Table 3:** Statistical analysis of Alpha diversity index.

Sample	Chao1	Simpson	Shannon	Pielou's evenness	Observed species	Faith's PD	Goods coverage
CZ1	776.424	0.910572	5.64342	0.588308	772.1	44.4865	0.999433
CZ2	301.297	0.655669	3.32679	0.406681	290.1	30.4710	0.999602
CZ3	456.026	0.881407	4.67368	0.53205	440.9	33.3046	0.999271
CZ4	525.863	0.782378	4.13132	0.458692	514.4	35.1551	0.999288
LZ1	182.193	0.204933	0.978133	0.132322	168.0	57.6407	0.999628
LZ2	97.6659	0.398078	1.464920	0.22345	94.1	15.9611	0.999873
LZ3	672.409	0.600411	3.21118	0.344443	640.4	38.3927	0.998668
LZ4	600.000	0.722918	3.59158	0.392133	571.7	35.7839	0.998896
AZ1	309.775	0.457996	2.07760	0.253912	290.5	25.4681	0.999414
AZ2	472.144	0.788589	3.72562	0.422906	448.7	34.7309	0.999080
AZ3	139.305	0.426415	1.52562	0.217816	128.4	39.1726	0.999709
AZ4	372.352	0.6599	3.01108	0.352894	370.3	27.8166	0.999680

### 3.4 Feeding sodium acetate/sodium butyrate changed microbiota of yaks in different taxon

**Fig. 5:**
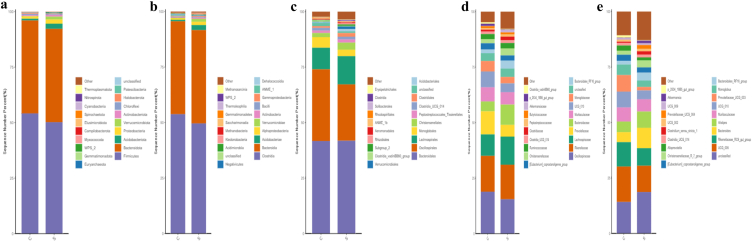
Effect of feeding SA/SB on microbiota abundance of ruminant calves in different taxa levels. (a) Phylum, (b) Class, (c) Order, (d) Family, (e) Genera.

Firmicutes and Bacteroidota were the core phyla in group C (54.15%, 41.87%) and S (50.20%, 42.05%) (Fig. 5a). At the class level, the considerable classes were Clostridia and Bacteroides in group C (53.75, 41.87%) and S (49.60%, 42.04%) (Fig. 5b). At the order level, the primary orders were Bacteroides, Oscillospirales and Lachnospirales in yak calves of group C (41.73%, 32.31%, 9.73%) and S (41.85%, 25.38%, 12.74%) (Fig. 5c). At the family level, Oscillospiraceae (18.89%), Rikenellaceae (16.17%), Prevotellaceae (10.40%), and Lachnospiraceae (9.62%) were mainly found in yak calves belonging to group C, while Oscillospiraceae (15.49%), Rikenellaceae (15.58%), Lachnospiraceae (12.63%), and Bacteroidaceae (9.17%) were primarily detected in group S (Fig. 5d). At the genera level, UCG_005 (153.97%), *Rikenellaceae*_RC9_gut_group (11.01%), *Prevotellaceae*_UCG_003 (7.47%) and UCG_010 (7.14%) were the prevailing genera in yak's calves of group C, while UCG_005 (11.87%), *Bacteroides* (9.17%), *Alistipes* (7.32%), and *Muribaculaceae* (5.54%) were mainly examined in yaks of group S (Fig. 5e).

**Fig. 6:**
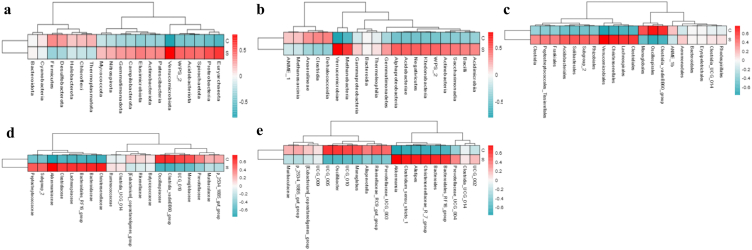
Grouping clustering heat map analysis of the effect of feeding sodium acetate/sodium butyrate on yak microbiota. (a) Phylum, (b) Class, (c) Order, (d) Family, (e) Genera.

Grouping clustering heat map analysis showed that the phyla of Proteobacteria, Verrucomicrobiota and Euryarchaeota were more abundant in group S (Fig. 6a). At the order level, the abundance of Verrucomicrobiae and Methanobacteria was higher in supplemented yak calves (Fig. 6b). At the class level, Oscillospirales, Monoglobales and Clostridia_vadinBB60_group were higher in group C, while Lachnospirales, Christensenellales, Clostridiales, Verrucomicrobiales and Solibacterales were higher in group S (Fig. 6c). At the family level, Oscillospiraceae, UCG_010, Monoglobaceae and Clostridia_vadinBB60_group were higher in yak calves of group C, while Lachnospiraceae, Bacteroidaceae, Bacteroidales_RF16_group, Christensenellaceae, Clostridiaceae and Akkermansiaceae were lower (Fig. 6d). At the genera level, UCG_005, UCG_010, *Monoglobus*, and *Oscillibacte*r were higher in yak of group C, while Bacteroides, Alistipes, Bacteroidales_RF16_group, Christensenellaceae_R_7_group, Clostridium_sensu_stricto_1 and Akkermansia were lower (Fig. 6e).

**Fig. 7:**
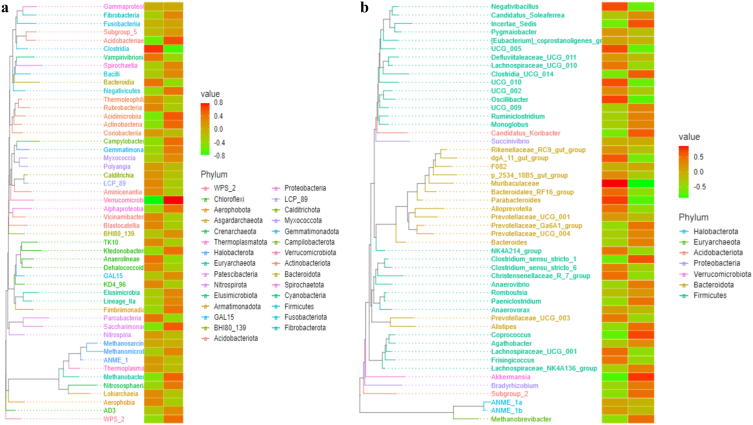
Effect of feeding SA/SB on yak microbiota in different class and genera levels by species evolutionary tree with heat map analysis. (a) Class, (b) Genera.

Species evolutionary tree with heat map analysis revealed that the abundance of classes of Methanobacteria, Saccharimonadia, Verrucomicrobiae, Acidimicrobiia and Acidobacteriae was lower in yaks of group C, while Clostridia was higher (Fig. 7a). At the genera level, the abundance of *Parabacteroides*, *Muribaculaceae*, dgA_11_gut_group, *Bacteroidales*_RF16_group, UCG_010, *Negativibacillus*, UCG_005, *Oscillibacter,* and *Lachnospiraceae*_UCG_001 were higher in yaks in group C, while *Methanobrevibacter*, Subgroup_2, *Akkermansia*, *Alistipes*, *Clostridium*_sensu_stricto_1, Incertae_Sedis, *Clostridia*_UCG_014, *Candidatus_Koribacter,* and *Coprococcus* were lower (Fig. 7b).

**Fig. 8:**
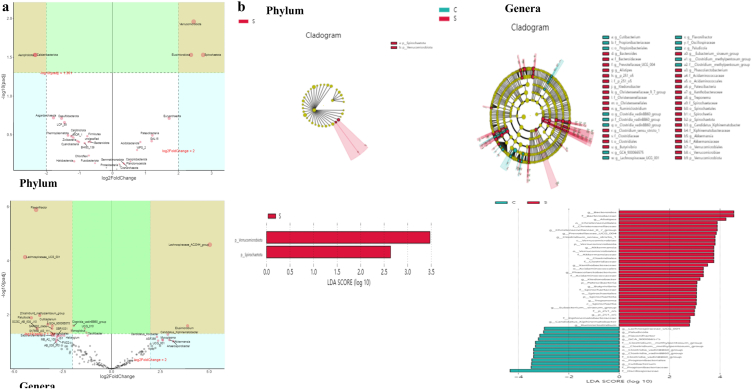
Different marker species analysis in yak calf's microbiota. (a) DESeq2Volcano Plots analysis, (b) LEfSe analysis.

To further uncover the distinguished microbiota between groups C and S, we performed DESeq2 Volcano Plots analysis and discovered that the abundance of Verrucomicrobiota (*p* < 0.001), Elusimicrobiota (*p* < 0.01), Spirochaetota (*p* < 0.01), and Euryarchaeota (*p* < 0.05) were significantly higher in yak calves of group S, while the phyla of Aerophobota (*p* < 0.01), Caldatribacteriota (*p* < 0.01), Asgardarchaeota (*p* < 0.05), and Desulfobacterota (*p* < 0.05) were lower (Fig. 8a). At the genera level, Lachnospiraceae_AC2044_group (*p <* 0.001), Elusimicrobium (*p <* 0.001), Candidatus_Xiphinematobacter (*p* < 0.01), Candidatus_Koribacter (*p* < 0.01), Mycoplasma (*p* < 0.01), Akkermansia (*p* < 0.01), UCG_001 (*p* < 0.05), ASF356 (*p* < 0.05), Anaerosporobacter (*p* < 0.05), Phenylobacterium (*p* < 0.05), p_251_o5 (*p* < 0.05), Methanobrevibacter (*p* < 0.05), Phascolarctobacterium (*p* < 0.05), [Eubacterium]_xylanophilum_group (*p* < 0.05), Candidatus_Saccharimonas (*p* < 0.05), Treponema (*p* < 0.05), Atopobium (*p* < 0.05), Oscillospira (*p* < 0.05) and [Eubacterium]_siraeum_group (*p* < 0.05) were markedly higher in group S, while Flavonifractor (*p <* 0.001), Lachnospiraceae_UCG_001 (*p <* 0.001), [Clostridium]_methylpentosum_group (*p <* 0.001), Paludicola (*p <* 0.001), Cutibacterium (*p <* 0.001), GCA_900066575 (*p <* 0.001), Clostridia_vadinBB60_group (*p* < 0.01), SCGC_AB_539_J10 (*p* < 0.01), SAR202_clade (*p* < 0.01), UCG_010 (*p* < 0.01), Monoglobus (*p* < 0.01), Aerophobales (*p* < 0.01), JS1 (*p* < 0.01), SBR1031 (*p* < 0.01), Sh765B_AG_111 (*p* < 0.01), Saccharofermentans (*p* < 0.01), Oscillibacter (*p* < 0.01), Haliangium (*p* < 0.01), MB_A2_108 (*p* < 0.01), FW22 (*p* < 0.01), AB_539_J10 (*p* < 0.05), Dehalococcoidia (*p* < 0.05), MSBL5 (*p* < 0.05), Achromobacter (*p* < 0.05), EMP_G18 (*p* < 0.05), SB_5 (*p* < 0.05), Dietzia (*p* < 0.05), Pygmaiobacter (*p* < 0.05), Lokiarchaeia (*p* < 0.05), DscP2 (*p* < 0.05), Prevotellaceae_UCG_003 (*p* < 0.05), Fournierella (*p* < 0.05), Aminicenantales (*p* < 0.05), GIF3 (*p* < 0.05), Nocardioides (*p* < 0.05) and [Eubacterium]_oxidoreducens_group were lower (Fig. 8a). LEfSe analysis confirmed the results found in DESeq2Volcano Plots analysis. At the phylum level, p__Verrucomicrobiota (*p* < 0.01) and p__Spirochaetota (*p* < 0.05) were significantly higher in animals in group S (Fig. 8b). At the genera level, the abundance of f__Akkermansiaceae (*p* < 0.01), o__Verrucomicrobiales (*p* < 0.01), p__Verrucomicrobiota (*p* < 0.01), g__Clostridium_sensu_stricto_1 (*p* < 0.01), g__Akkermansia (*p* < 0.01), g__Alistipes (*p* < 0.01), g__Butyrivibrio (*p* < 0.05), g___Eubacterium__siraeum_group (*p* < 0.05), g__Christensenellaceae_R_7_group (*p* < 0.05), g__Phascolarctobacterium (*p* < 0.05), f__Bacteroidaceae (*p* < 0.05), g__Candidatus_Xiphinematobacter (*p* < 0.05), g__Xanthobacteraceae (*p* < 0.05), f__Clostridiaceae (*p* < 0.05), g__Treponema (*p* < 0.05), g__p_251_o5 (*p* < 0.05), g__Bacteroides (*p* < 0.05), g__Ruminiclostridium (*p* < 0.05), f__Spirochaetaceae (*p* < 0.05), f__Christensenellaceae (*p* < 0.05), o__Acidaminococcales (*p* < 0.05), f__p_251_o5 (*p* < 0.05), o__Christensenellales (*p* < 0.05), p__Patescibacteria (*p* < 0.05), f__Xiphinematobacteraceae (*p* < 0.05), g__Ktedonobacter (*p* < 0.05), p__Spirochaetota (*p* < 0.05), o__Clostridiales (*p* < 0.05), f__Acidaminococcaceae (*p* < 0.05), c__Spirochaetia (*p* < 0.05), g__Prevotellaceae_UCG_004 (*p* < 0.05), o__Spirochaetales (*p* < 0.05) and c__Verrucomicrobiae (*p* < 0.05) were significantly higher in yaks of group S, while g__Flavonifractor (*p* < 0.01), g__Lachnospiraceae_UCG_001 (*p* < 0.01), f___Clostridium__methylpentosum_group (*p* < 0.05), f__Oscillospiraceae (*p* < 0.05), g__Paludicola (*p* < 0.05), f__Clostridia_vadinBB60_group (*p* < 0.05), g__GCA_900066575 (*p* < 0.05), g__Clostridia_vadinBB60_group (*p* < 0.05), g___Clostridium__methylpentosum_group (*p* < 0.05), o__Propionibacteriales (*p* < 0.05), o__Clostridia_vadinBB60_group (*p* < 0.05), f__Propionibacteriaceae (*p* < 0.05) and g__Cutibacterium (*p* < 0.05) were significantly lower (Fig. 8b).

### 3.5 SA/SB supplementation affected the function of gut microbiota of yak calves

**Fig. 9:**
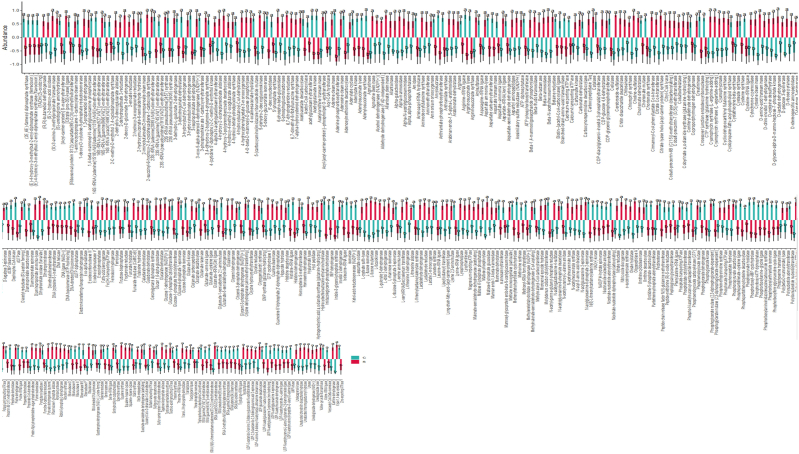
Feeding SA/SB affected the enzyme abundance of gut microbiota of yak calves.

**Fig. 10:**
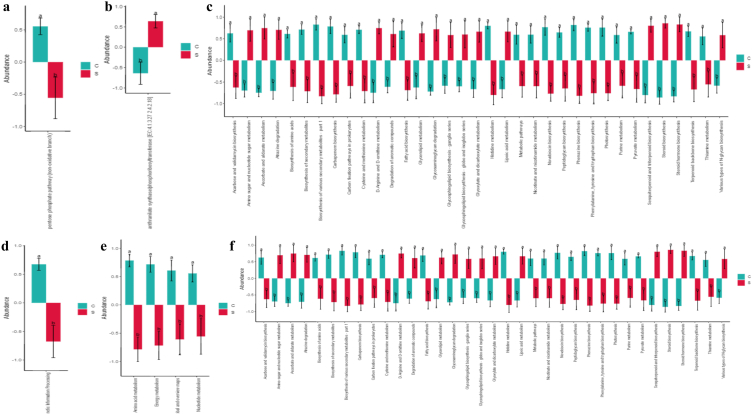
Supplementation of SA/SB affected the function of gut microbiota of yak calves. (a) MetaCyc, (b) KEGG Orthology, (c) KEGG, (d) KEGG L1, (e) KEGG L2, (F) KEGG L3.

Among the 1948 enzymes, the abundance of 432 enzymes was obviously different between yak groups, with 253 higher and 179 lower abundant enzymes in group S (Fig. 9). MetaCyc pathway abundance analysis found pentose phosphate pathway (non-oxidative branch) was significantly lower in yaks from group S (*p* < 0.05) (Fig. 10a). KEGG orthology analysis showed that the abundance of anthranilate synthase/phosphoribosyl transferase [EC: 4.1.3.27 2.4.2.18] was significantly higher in group S (*p* < 0.05) (Fig. 10b). The KEGG analysis discovered that genetic information processing pathways of Aminoacyl-tRNA biosynthesis (*p* < 0.05), DNA replication (*p* < 0.05), Homologous recombination (*p* < 0.05), Mismatch repair (*p* < 0.05), Nucleotide excision repair (*p* < 0.05), Protein export (*p* < 0.05), Protein processing in the endoplasmic reticulum (*p* < 0.05), and Ribosome (*p* < 0.05) were significantly higher in animals of group C. The abundance of metabolism pathways of Acarbose and validamycin biosynthesis (*p* < 0.05), Biosynthesis of amino acids (*p* < 0.05), Biosynthesis of secondary metabolites (*p* < 0.05), Biosynthesis of various secondary metabolites - part 1 (*p* < 0.05), Carbapenem biosynthesis (*p* < 0.05), Carbon fixation pathways in prokaryotes (*p* < 0.05), Cysteine and methionine metabolism (*p* < 0.05), Fatty acid biosynthesis (*p* < 0.05), Histidine metabolism (*p* < 0.05), Metabolic pathways (*p* < 0.05), Nicotinate and nicotinamide metabolism (*p* < 0.05), Novobiocin biosynthesis (*p* < 0.05), Peptidoglycan biosynthesis (*p* < 0.05), Phenazine biosynthesis (*p* < 0.05), Phenylalanine, tyrosine and tryptophan biosynthesis (*p* < 0.05), Photosynthesis (*p* < 0.05), Purine metabolism (*p* < 0.05), Pyruvate metabolism (*p* < 0.05), Terpenoid backbone biosynthesis (*p* < 0.05) and Thiamine metabolism (*p* < 0.05) were significantly higher in group C, while Amino sugar and nucleotide sugar metabolism (*p* < 0.05), Ascorbate and aldarate metabolism (*p* < 0.05), Atrazine degradation (*p* < 0.05), d-Arginine and d-ornithine metabolism (*p* < 0.05), Degradation of aromatic compounds (*p* < 0.05), Glycerolipid metabolism (*p* < 0.05), Glycosaminoglycan degradation (*p* < 0.05), Glycosphingolipid biosynthesis - ganglio series (*p* < 0.05), Glycosphingolipid biosynthesis - globo and isoglobo series (*p* < 0.05), Glyoxylate and dicarboxylate metabolism (*p* < 0.05), Lipoic acid metabolism (*p* < 0.05), Sesquiterpenoid and triterpenoid biosynthesis (*p* < 0.05), Steroid biosynthesis (*p* < 0.05), Steroid hormone biosynthesis (*p* < 0.05) and Various types of N-glycan biosynthesis (*p* < 0.05) were obviously higher in group S (Fig. 10c). KEGG L1 analysis showed that the genetic information processing pathway in group C was significantly higher than that in SA and SB treated calves (Fig. 10d). KEGG L2 discovered that pathways of Amino acid metabolism (*p* < 0.05), Energy metabolism (*p* < 0.05), Folding, sorting, and degradation (*p* < 0.05) and Translation (*p* < 0.05) were memorably higher in calves in group C, while Cancer overview (*p* < 0.05), Environmental adaptation (*p* < 0.05), Immune disease (*p* < 0.05), Membrane transport (*p* < 0.05), Nucleotide metabolism (*p* < 0.05) and Transport and catabolism (*p* < 0.05) were dramatically higher in ruminants in SA and SB treated yaks (Fig. 10e). KEGG L3 uncovered those 66 differently abundant pathways in both groups, with 39 higher and 27 lower abundant pathways in group C (Fig. 10f).

## 4 Discussion

**Fig. 11:**
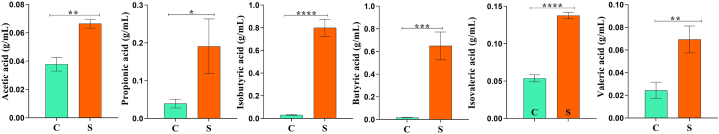
Supplementation of SA/SB increased SCFAs in yaks.

Calf diarrhea is commonly recognized as a distressing condition which has harmful effects on livestock industry [36,37]. New therapeutic approaches not only safeguard animal health but also improve livestock production along with breeding benefits [5,38]. We found a lower diarrhea score in yak calves supplemented with sodium acetate and sodium butyrate. Furthermore, higher weekly weight gains and net weekly weight gains were recorded in yaks belonging to group S, which demonstrated that SA/SB can prevent diarrhea in yak calves. Reactive oxygen species are commonly known for disrupting macromolecules, including intracellular proteins, lipids, and nucleic acid, which may negatively affect metabolism and physiology [39]. GSH-px, T-AOC, and SOD are important antioxidant enzymes against ROS [[39], [40], [41]]. The higher activity of these enzymes in group S yaks indicated that SA/SB increased oxidation resistance in calves. MDA is a biomarker of oxidative stress [42], the lower levels of MDA in yak calves fed with SA/SB showed lower lipid peroxidation in animals. The free radical NO can lead to endothelium damage by reacting with superoxide and creating toxic peroxynitrites [43]. Previous study showed that NO was highly related to inflammatory processes [44]. The lower concentration of NO in group S revealed that SA/SB could decrease inflammatory response and endothelium injury by scavenging NO free radicals. TNF-α is a cytokine that involves in inflammatory pathogenesis [45]; IL-1β is a strong proinflammatory cytokine, which can employ various effects on cells and cause severe inflammatory reactions [46]. The lower levels of these two inflammatory factors in yaks in groups S indicated that SA and SB could reduce intestinal inflammation in yak calves. The concentration of SCFAs in groups S was obviously higher than in control yaks (Fig. 11), which was in line with our previous results in mice [24,47].

To further reveal the effect of SA/SB on the microbiota of yaks, we performed high throughout sequencing. Almost 113 000 filtered sequences were achieved in both groups and aligned to 7806 ASVs in yaks. SA and SB supplementation increased the species diversity of ruminants in group S by increasing the simpson index. SA/SB also changed the microbiota structure, which was revealed by beta diversity analysis. The change in microbiota structure was also confirmed by microbiota abundance analysis of yak calves at different taxa levels, grouping clustering heat map analysis and species evolutionary tree with heat map analysis. Microbiota change affected its function with 432 different abundant enzymes and one different MetaCyc pathway between yak groups. Similarly, SA and SB affected KEGG function and its related pathways at different levels.

Finally, we explored the marker bacteria between yaks in groups C and S, and we found 8 phyla and 54 genera were significantly different between two groups. Among those genus, lower abundance of *Lachnospiraceae*_AC2044_group and p_251_o5 was reported in diarrheal horses [48], Elusimicrobium in Type 2 diabetes [49], Methanobrevibacter in cirrhotic patients [50], [Eubacterium]_xylanophilum_group in intestinal injury mice induced by radiation [51], Candidatus_Saccharimonas in colitis mice [52], Atopobium in Chlamydia trachomatis infected women with infertility [53], and [Eubacterium]_siraeum_group in neurobehavioral injury mice induced by lead [15]. The higher abundance of these genera in group S may contribute to a decrease in diarrhea in yaks. Akkermansia and Oscillospira are novel probiotics, Akkermansia is an important genus in preventing disease development [54], while Oscillospira is producing SCFAs [55]. ASF356, Anaerosporobacter, and Phascolarctobacterium are SCFAs generating genera [56], and a lower abundance of ASF356 and Phascolarctobacterium is reported in immunosuppressed mice [57] and IBD rats [58]. The higher abundance of these genera in group S also explained the higher concentration of SCFAs and lower diarrhea in yak calves. Flavonifractor is a pro-inflammatory genus [59]; the lower abundance of bacteria means SA and SB supplementation decreased the inflammatory response in calves in group S. Oscillibacter is a harmful bacterium and positively related to pro-inflammatory cytokines [60]. The lower abundance of this genera in ruminant calves indicated that SA/SB relieves diarrhea by decreasing the inflammatory response in calves in groups S. The higher abundance of Fournierella was reported in *Salmonella typhimurium* infected ducks, and this genus could enhance intestinal inflammation [61]. The less abundance of Fournierella in yaks of group S further demonstrated that SA/SB could decrease inflammatory reactions in yaks. Achromobacter is an antibiotic-resistant pathogenic genus [62]. The lower abundance of this genus in yaks in group S revealed that SA/SB can inhibit harmful bacteria in calves. Previously, a higher abundance of Lachnospiraceae_UCG_001, Cutibacterium, Monoglobus, Pygmaiobacter, and Prevotellaceae_UCG_003 was found in colitis mice [63], people with cancer pain [2], diabetic rats [64], and diarrhea in yaks [7,65], whereas the lower abundance of these genus in group S indicates a lower diarrhea rate in yaks.

## 5 Conclusion

In conclusion, our study demonstrated that SA and SB could decrease diarrhea rates in yak calves on the plateau via increasing antioxidant ability and SCFAs, while decreasing inflammatory responses in yaks by modulating gut microbiota. The current results provide new insights for the prevention and treatment of diarrhea in yaks.

## Data available statement

Sequences data from yak calves were stored in the NCBI database under accession number: PRJNA978566.

## Ethics statement

All the experiment operations were according to the instructions and approval of Laboratory Animals Research Centre of Tibet Agriculture and Animal Husbandry College and the ethics committee of Nanjing Agricultural University (NJAU.No20220520108).

## CRediT authorship contribution statement

**Qinghui Kong:** Writing – review & editing, Writing – original draft, Validation, Software, Methodology, Investigation, Data curation, Conceptualization. **Xiushuang Chen:** Writing – original draft, Resources, Methodology, Funding acquisition, Formal analysis, Data curation. **Yang Liu:** Writing – review & editing, Validation, Supervision, Funding acquisition, Conceptualization. **Farah Ali:** Writing – review & editing, Validation, Formal analysis, Data curation. **Asif Idrees:** Writing – review & editing, Software, Formal analysis, Data curation. **Farid Shokry Ataya:** Writing – review & editing, Resources, Funding acquisition, Formal analysis, Data curation. **Zhenda Shang:** Writing – review & editing, Writing – original draft, Visualization, Supervision, Resources, Project administration, Investigation, Conceptualization. **Kun Li:** Writing – original draft, Visualization, Validation, Software, Resources, Project administration, Investigation, Funding acquisition, Data curation, Conceptualization.

## Declaration of competing interest

The authors declare that they have no known competing financial interests or personal relationships that could have appeared to influence the work reported in this paper.

## References

[1] Chang M.N., Wei J.Y., Hao L.Y., Ma F.T., Li H.Y., Zhao S.G., Sun P. (2020). Effects of different types of zinc supplement on the growth, incidence of diarrhea, immune function, and rectal microbiota of newborn dairy calves. J. Dairy Sci..

[2] Wang H., Luo J., Chen X., Hu H., Li S., Zhang Y., Shi C. (2022). Clinical observation of the effects of oral opioid on inflammatory cytokines and gut microbiota in patients with moderate to severe cancer pain: a retrospective cohort study. Pain and Therapy.

[3] Wang S., Cao Z., Wu Q., Ai M.H.A., Dong H. (2023). A comparative analysis and verification of differentially expressed miRNAs could provide new insights for the treatment of endometritis in yaks. Pak. Vet. J..

[4] Li K., Zeng Z., Liu J., Pei L., Wang Y., Li A., Kulyar M.F., Shahzad M., Mehmood K., Li J., Qi D. (2022). Effects of short-chain fatty acid modulation on potentially diarrhea-causing pathogens in yaks through metagenomic sequencing. Front. Cell. Infect. Microbiol..

[5] Li K., Mehmood K., Zhang H., Jiang X., Shahzad M., Dong X., Li J. (2018). Characterization of fungus microbial diversity in healthy and diarrheal yaks in Gannan region of Tibet Autonomous Prefecture. Acta Trop..

[6] Li Y., Xia S., Jiang X., Feng C., Gong S., Ma J., Fang Z., Yin J., Yin Y. (2021). Gut microbiota and diarrhea: an updated review. Front. Cell. Infect. Microbiol..

[7] Dong H., Liu B., Li A., Iqbal M., Mehmood K., Jamil T., Chang Y., Zhang H., Wu Q. (2021). Microbiome analysis reveals the attenuation effect of lactobacillus from yaks on diarrhea via modulation of gut microbiota. Front. Cell. Infect. Microbiol..

[8] Humayun A., Zafar M.A., Yousaf A., Hasan M.U. (2022). Resuscitative effects of hyperosmotic sodium bicarbonate on strong ion metabolic acidosis in salmonella-induced neonatal calf diarrhea in buffalo calves. Pak. Vet. J..

[9] Ren Y., Zhaxi Y., Liu M., Idrees A., Li K. (2023). Revealing the fungi microbiome difference of suffolk cross with Tibetan sheep on plateau. Pak. Vet. J..

[10] Chung J.J., Rayburn M.C., Chigerwe M. (2019). Randomized controlled clinical trial on the effect of oral immunoglobulin supplementation on neonatal dairy calves with diarrhea. J. Vet. Intern. Med..

[11] Chen X., Saeed N.M., Ding J., Dong H., Kulyar M.F.E.A., Bhutta Z.A., Mehmood K., Ali M.M., Irshad I., Zeng J., Liu J., Wu Q., Li K. (2022). Molecular epidemiological investigation of Cryptosporidium sp., Giardia duodenalis, Enterocytozoon bieneusi and Blastocystis sp. infection in free-ranged yaks and Tibetan pigs on the plateau. Pak. Vet. J..

[12] Kim H.S., Whon T.W., Sung H., Jeong Y., Jung E.S., Shin N., Hyun D., Kim P.S., Lee J., Lee C.H., Bae J. (2021). Longitudinal evaluation of fecal microbiota transplantation for ameliorating calf diarrhea and improving growth performance. Nat. Commun..

[13] Gomez D.E., Arroyo L.G., Costa M.C., Viel L., Weese J.S. (2017). Characterization of the fecal bacterial microbiota of healthy and diarrheic dairy calves. J. Vet. Intern. Med..

[14] Han Z., Li K., Shahzad M., Zhang H., Luo H., Qiu G., Lan Y., Wang X., Mehmood K., Li J. (2017). Analysis of the intestinal microbial community in healthy and diarrheal perinatal yaks by high-throughput sequencing. Microb. Pathog..

[15] Li Y., Lan Y., Zhang S., Wang X. (2022). Comparative analysis of gut microbiota between healthy and diarrheic horses. Front. Vet. Sci..

[16] Liu J., Wang X., Zhang W., Kulyar M.F., Ullah K., Han Z., Qin J., Bi C., Wang Y., Li K. (2022). Comparative analysis of gut microbiota in healthy and diarrheic yaks. Microb. Cell Factories.

[17] Adak A., Khan M.R. (2019). An insight into gut microbiota and its functionalities. Cell. Mol. Life Sci..

[18] Chen L., Wang J. (2022). Gut microbiota and inflammatory bowel disease. WIREs Mechanisms of Disease.

[19] Becattini S., Taur Y., Pamer E.G. (2016). Antibiotic-induced changes in the intestinal microbiota and disease. Trends Mol. Med..

[20] Liu P., Wang Y., Yang G., Zhang Q., Meng L., Xin Y., Jiang X. (2021). The role of short-chain fatty acids in intestinal barrier function, inflammation, oxidative stress, and colonic carcinogenesis. Pharmacol. Res..

[21] Macfarlane S., Macfarlane G.T. (2003). Regulation of short-chain fatty acid production. Proc. Nutr. Soc..

[22] Li Y., Liu A., Chen L., Xiang Y., Huang D., Huang W., Chen Z., Fan H., Meng X. (2022). Lactobacillus plantarum WSJ-06 alleviates neurobehavioral injury induced by lead in mice through the gut microbiota. Food Chem. Toxicol..

[23] Dong H., Chen X., Zhao X., Zhao C., Mehmood K., Kulyar M.F., Bhutta Z.A., Zeng J., Nawaz S., Wu Q., Li K. (2023). Intestine microbiota and SCFAs response in naturally Cryptosporidium-infected plateau yaks. Front. Cell. Infect. Microbiol..

[24] Chen X., Kong Q., Zhao X., Zhao C., Hao P., Irshad I., Lei H., Kulyar M.F., Bhutta Z.A., Ashfaq H., Sha Q., Li K., Wu Y. (2022). Sodium acetate/sodium butyrate alleviates lipopolysaccharide-induced diarrhea in mice via regulating the gut microbiota, inflammatory cytokines, antioxidant levels, and NLRP3/Caspase-1 signaling. Front. Microbiol..

[25] Callahan B.J., Mcmurdie P.J., Rosen M.J., Han A.W., Johnson A.J.A., Holmes S.P. (2016). DADA2: high-resolution sample inference from Illumina amplicon data. Nat. Methods.

[26] Bokulich N.A., Kaehler B.D., Rideout J.R., Dillon M., Bolyen E., Knight R., Huttley G.A., Gregory Caporaso J. (2018). Optimizing taxonomic classification of marker-gene amplicon sequences with QIIME 2's q2-feature-classifier plugin. Microbiome.

[27] Kumar K., Cava F. (2018). Principal coordinate analysis assisted chromatographic analysis of bacterial cell wall collection: a robust classification approach. Anal. Biochem..

[28] Vazquez-Baeza Y., Pirrung M., Gonzalez A., Knight R. (2013). EMPeror: a tool for visualizing high-throughput microbial community data. GigaScience.

[29] Petersen K.S., Anderson S., Chen See J.R., Leister J., Kris-Etherton P.M., Lamendella R. (2022). Herbs and spices modulate gut bacterial composition in adults at risk for CVD: results of a prespecified exploratory analysis from a randomized, crossover, controlled-feeding study. J. Nutr..

[30] Estavoyer M., François O. (2022). Theoretical analysis of principal components in an umbrella model of intraspecific evolution. Theor. Popul. Biol..

[31] Chen X., Zhao X., Zhao C., Ashfaq H., Fakhar-E-Alam Kulyar M., Bhutta Z.A., Ali M.M., Mansoor M.K., Li K. (2023). Cryptosporidium infection induced the dropping of SCFAS and dysbiosis in intestinal microbiome of Tibetan pigs. Microb. Pathog..

[32] Ding X., Jiang H., Zhang R., Chen X., Yu H., Zu Y., Tan S., Wang X., Wang Q., Xu W., Fouad D., Saleem M.U., Liu Z. (2023). Comparative analysis of nasal microbial community between Tibetan sheep with different ages. Pak. Vet. J..

[33] Shanmugam G., Lee S.H., Jeon J. (2021). EzMAP: easy microbiome analysis platform. BMC Bioinf..

[34] Langille M.G.I., Zaneveld J., Caporaso J.G., Mcdonald D., Knights D., Reyes J.A., Clemente J.C., Burkepile D.E., Vega Thurber R.L., Knight R., Beiko R.G., Huttenhower C. (2013). Predictive functional profiling of microbial communities using 16S rRNA marker gene sequences. Nat. Biotechnol..

[35] Zhang S., Wang H., Zhu M. (2019). A sensitive GC/MS detection method for analyzing microbial metabolites short chain fatty acids in fecal and serum samples. Talanta.

[36] Lei W., Sijia L., Wen Z., Sun N., Saleem M.U., Nazar M., Hassan M.F., Ataya F.S., Qinghui K., Kun L. (2023). The effects of Cryptosporidium infection on gut fungi and enzyme abundance in *Sus domesticus*. Asian J. Agric. Biol..

[37] Basit M.A., Arifah A.K., Chwen L.T., Salleh A., Kaka U., Idris S.B., Farooq A.A., Javid M.A., Murtaza S. (2023). Qualitative and quantitative phytochemical analysis, antioxidant activity and antimicrobial potential of selected herbs Piper betle and Persicaria odorata leaf extracts. Asian J Agric Biol.

[38] Okeniyi F.A., Oghenochuko O.M., Olawoye S.O., Animashahun R.A., Adeyonu A.G., Akpor O.B. (2022). Antimicrobial potentials of mucus mucin from different species of giant African land snails on some typed culture pathogenic bacteria. Asian J Agric Biol.

[39] Yang S., Lian G. (2020). ROS and diseases: role in metabolism and energy supply. Mol. Cell. Biochem..

[40] Fang Yukun, Xing Chenghong, Wang Xiaoyu, Cao Huabin, Zhang Caiying, Guo Xiaoquan, Zhuang Yu, Hu Rui-Ming, Hu Guoliang, Fan Yang (2021). Activation of the ROS/HO-1/NQO1 signaling pathway contributes to the copper-induced oxidative stress and autophagy in duck renal tubular epithelial cells. Sci. Total Environ..

[41] Qin S., She F., Zhao F., Li L., Chen F. (2022). Selenium-chitosan alleviates the toxic effects of Zearalenone on antioxidant and immune function in mice. Front. Vet. Sci..

[42] Tsikas D. (2017). Assessment of lipid peroxidation by measuring malondialdehyde (MDA) and relatives in biological samples: analytical and biological challenges. Anal. Biochem..

[43] Zhang Z., Zhang Q., Xue Y., Chen G., Wu Z., Fang H. (2019). Serum levels of total antioxidant status, nitric oxide and nitric oxide synthase in minor recurrent aphthous stomatitis patients. Medicine.

[44] Yapışlar H., Aydogan S., Borlu M., Ascioglu Ö. (2007). Decreased nitric oxide and increased platelet aggregation levels in patients with Behçet's disease. Thromb. Res..

[45] Jang D., Lee A., Shin H., Song H., Park J., Kang T., Lee S., Yang S. (2021). The role of tumor necrosis factor alpha (TNF-α) in autoimmune disease and current TNF-α inhibitors in therapeutics. Int. J. Mol. Sci..

[46] Wang Y., Che M., Xin J., Zheng Z., Li J., Zhang S. (2020). The role of IL-1β and TNF-α in intervertebral disc degeneration. Biomed. Pharmacother..

[47] Jabeen Z., Bukhari S.A., Malik S.A., Hussain G., Kamal S. (2023). Improved gut microbiota escalates muscle function rehabilitation and ameliorates oxidative stress following mechanically induced peripheral nerve injury in mice. Pak. Vet. J..

[48] Li A., Wang Y., Wang Y., Dong H., Wu Q., Mehmood K., Chang Z., Chang Y., Shi L., Tang Z., Zhang H. (2021). Microbiome analysis reveals soil microbial community alteration with the effect of animal excretion contamination and altitude in Tibetan Plateau of China. International Soil and Water Conservation Research.

[49] Almugadam B.S., Liu Y., Chen S., Wang C., Shao C., Ren B., Tang L., Hatziagelaki E. (2020). Alterations of gut microbiota in type 2 diabetes individuals and the confounding effect of antidiabetic agents. J. Diabetes Res..

[50] Ponziani F.R., Picca A., Marzetti E., Calvani R., Conta G., Del Chierico F., Capuani G., Faccia M., Fianchi F., Funaro B., Josè Coelho Junior H., Petito V., Rinninella E., Paroni Sterbini F., Reddel S., Vernocchi P., Cristina Mele M., Miccheli A., Putignani L., Sanguinetti M., Pompili M., Gasbarrini A., Hernandez Gea V. (2021). Characterization of the gut‐liver‐muscle axis in cirrhotic patients with sarcopenia. Liver Int..

[51] Zhao T., Xie L., Cai S., Xu J., Zhou H., Tang L., Yang C., Fang S., Li M., Tian Y. (2021). Dysbiosis of gut microbiota is associated with the progression of radiation-induced intestinal injury and is alleviated by oral compound probiotics in mouse model. Front. Cell. Infect. Microbiol..

[52] Chang F., He S., Dang C. (2022). Assisted selection of biomarkers by linear discriminant analysis effect size (LEfSe) in microbiome data. J. Vis. Exp..

[53] Chen H., Wang L., Zhao L., Luo L., Min S., Wen Y., Lei W., Shu M., Li Z. (2021). Alterations of vaginal microbiota in women with infertility and Chlamydia trachomatis infection. Front. Cell. Infect. Microbiol..

[54] Kalia V.C., Gong C., Shanmugam R., Lin H., Zhang L., Lee J. (2022). The emerging biotherapeutic agent: Akkermansia. Indian J. Microbiol..

[55] Yang J., Li Y., Wen Z., Liu W., Meng L., Huang H. (2021). Oscillospira - a candidate for the next-generation probiotics. Gut Microb..

[56] Tremlett H., Zhu F., Arnold D., Bar Or A., Bernstein C.N., Bonner C., Forbes J.D., Graham M., Hart J., Knox N.C., Marrie R.A., Mirza A.I., O Mahony J., Van Domselaar G., Yeh E.A., Zhao Y., Banwell B., Waubant E. (2021). The gut microbiota in pediatric multiple sclerosis and demyelinating syndromes. Annals of Clinical and Translational Neurology.

[57] Zhang J., Zhou H.C., He S.B., Zhang X.F., Ling Y.H., Li X.Y., Zhang H., Hou D.D. (2021). The immunoenhancement effects of sea buckthorn pulp oil in cyclophosphamide-induced immunosuppressed mice. Food Funct..

[58] Zhou F., Jiang H., Kong N., Lin J., Zhang F., Mai T., Cao Z., Xu M. (2022). Electroacupuncture attenuated anxiety and depression-like behavior via inhibition of hippocampal inflammatory response and metabolic disorders in TNBS-induced IBD rats. Oxid. Med. Cell. Longev..

[59] Eicher T.P., Mohajeri M.H. (2022). Overlapping mechanisms of action of brain-active bacteria and bacterial metabolites in the pathogenesis of common brain diseases. Nutrients.

[60] Huang P., Jiang A., Wang X., Zhou Y., Tang W., Ren C., Qian X., Zhou Z., Gong A. (2021). NMN maintains intestinal homeostasis by regulating the gut microbiota. Front. Nutr..

[61] Hao G., Li P., Huang J., Cui K., Liang L., Lin F., Lu Z., Sun S. (2023). Research Note: therapeutic effect of a Salmonella phage combination on chicks infected with Salmonella Typhimurium. Poultry Sci..

[62] Isler B., Kidd T.J., Stewart A.G., Harris P., Paterson D.L. (2020). Achromobacter infections and treatment options. Antimicrob. Agents Chemother..

[63] Huangfu L., Cai X., Yang J., Wang H., Li Y., Dai Z., Yang R., Lin X. (2021). Irisin attenuates inflammation in a mouse model of ulcerative colitis by altering the intestinal microbiota. Exp. Ther. Med..

[64] Zhang F., Chen D., Zhang L., Zhao Q., Ma Y., Zhang X., Zhao S., Chen C. (2022). Diaphragma juglandis extracts modifies the gut microbiota during prevention of type 2 diabetes in rats. J. Ethnopharmacol..

[65] Wu Z., Wei R., Tan X., Yang D., Liu D., Zhang J., Wang W. (2022). Characterization of gut microbiota dysbiosis of diarrheic adult yaks through 16S rRNA gene sequences. Front. Vet. Sci..

